# Cucumber CsTRY Negatively Regulates Anthocyanin Biosynthesis and Trichome Formation When Expressed in Tobacco

**DOI:** 10.3389/fpls.2019.01232

**Published:** 2019-10-08

**Authors:** Leyu Zhang, Jian Pan, Gang Wang, Hui Du, Huanle He, Junsong Pan, Run Cai

**Affiliations:** ^1^School of Agriculture and Biology, Shanghai Jiao Tong University, Shanghai, China; ^2^State Key Laboratory of Vegetable Germplasm Innovation, Tianjin, China

**Keywords:** cucumber, trichome, anthocyanin, *CsTRY*, *CsMYB6*, tobacco

## Abstract

The development of trichomes (spines) on cucumber fruits is an important agronomic trait. It has been reported that two MYB family members, *CsMYB6* (*Csa3G824850*) and *CsTRY* (*Csa5G139610*) act as negative regulators of trichome or fruit spine initiation. To further study the functions of these two genes, we overexpressed them in tobacco, and found that the flowers and seed coats of transformants overexpressing *CsTRY* displayed an unexpected defect in pigmentation that was not observed in plants overexpressing *CsMYB6*. Moreover, the expression of key genes in the flavonoid synthesis pathway was repressed in *CsTRY* overexpressing plants, which resulted in the decrease of several important flavonoid secondary metabolites. In addition, CsTRY could interact with the AN1 homologous gene CsAN1 (Csa7G044190) in cucumber, which further confirmed that CsTRY not only regulates the development of fruit spines, but also functions in the synthesis of flavonoids, acting as the repressor of anthocyanin synthesis.

## Introduction

Cucumber (*Cucumis sativus* L.) is a horticultural crop that is consumed worldwide (Huang et al., 2009; Yundaeng et al., 2015), and trichomes (spines) on the fruit are considered as an important commodity trait (Zhang et al., 2010; Yang et al., 2014; Li et al., 2015; Pan et al., 2015). The cucumber fruit, a pepo that develops from the ovary and receptacle, is covered with a thick cuticle, tubercules and trichomes (spines) (Roth, 1977; Wang et al., 2015). In the model plant *Arabidopsis thaliana*, trichome developments is initiated by a ternary complex (*GL1-GL3/EGL1-TTG1*) from epidermal cells, which leads to the expression of *GL2* and *TRY* (Oppenheimer et al., 1991; Galway et al., 1994; Walker, 1999; Payne et al., 2000; Szymanski et al., 2000; Larkin et al., 2003; Zhang et al., 2003). The TRY protein moves into neighboring cells, where it competes with *GL1* for binding to *GL3/EGL3* and prevents differentiation of the cells into trichomes (Schellmann et al., 2002; Esch et al., 2003; Zhang et al., 2003). Further, the transcriptional complex can also regulate anthocyanin biosynthesis genes to mediate anthocyanin biosynthesis, including NADPH-dependent dihydroflavonol reductase (DFR), leucoanthocyanidin dioxygenase (LDOX), and UDP-Glc:flavonoid 3′- O-glucosyltransferase (UF3GT). In *Artemisia annua*, an MYB family member, *AaMIXTA1*, can promotes trichome development and regulates cuticle biosynthesis. These reports suggest that, the genes that regulate trichome developments usually function in secondary metabolite biosynthesis (Yan et al., 2018).

In cucumber, *csgl1*/*mict*/*tbh* mutants produced microtrichomes, which means the *GL1* gene may be related to the development of trichomes. *csgl3*/*tril* mutants have a hairless phenotype, which means the *GL3* gene is related to the initiation of trichomes (Chen et al., 2014; Li et al., 2015; Pan et al., 2015; Zhao et al., 2015; Wang et al., 2016), and *CsTTG* is involved in the formation of fruit warts (Chen et al., 2016). It has been reported that *CsMYB6* and *CsTRY* act as negative regulators of trichome initiation, and they can reduce cucumber trichome density (Yang et al., 2018). However, the specific regulatory mechanisms are still unclear.

In this study, we overexpressed *CsTRY* and *CsMYB6* in tobacco (*Nicotiana tabacum L.*) and found the flowers and seed coats of *CsTRY* overexpressing transformants displayed an unexpected defect in pigmentation that was not found in *CsMYB6* overexpressing plants. Furthermore, the expression of key genes in the flavonoid synthesis pathway were repressed in *CsTRY* overexpressing plants. In addition, we determined the compound content in the anthocyanin synthesis pathway by LC-MS and found that the content of peonidin and several important flavonoid secondary metabolites was significantly decreased, which is consistent with the gene expression change. *CsTRY* could interact with the *AN1* homologous gene in cucumber. These results suggested that *CsTRY* not only regulates the development of fruit spines, but also functions in synthesis of flavonoids, acting as the repressor of anthocyanin synthesis.

## Materials and Methods

### *CsTRY* and *CsMYB6* Construct and Plant Transformation

The full-length *CsTRY* and *CsMYB6* coding region was amplified and inserted in the SacI and PstI sites of the pCambia2300 vector, containing the CaMV 35S promoter. The *CsMYB6* and *CsTRY* overexpression constructs were used for tobacco transformation (Horsch et al., 1985). The primes used are TRY-F(ATGGACAATCATCGT), TRY-R(TCATCCTCTTCTTCT), MYB6-F(ATGGGAAGGTCTCCT), MYB6-R(TCAGAATCTCAGGAA).

### Preparation of Nucleic Acids and cDNA Synthesis

Genomic DNA was isolated from *N. tabacum* young leaf material using a DNeasy plant mini kit (QIAGEN). Total RNAs were extracted from *N. tabacum* petal material of the wild-type and transgenic plants, respectively, using an RNA extraction kit (TRIzol Reagent, Invitrogen). First-strand cDNA was synthesized from 2 μg total RNA in a 20 μl reaction mixture with 0.5 μg oligo(dT)_15_, 0.75 mM dNTPs, 10 mM dithiothreitol (DTT), and 100 U SuperScript II RNase H-reverse transcriptase (Invitrogen).

### Semi-Quantitative RT-PCR and Real-Time RT-PCR Analysis

For expression analysis of different structural flavonoid genes, the petunia genes of interest, *PhCHS*, *PhCHI*, *PhF3H*, *PhF3’H*, *PhDFR*, and *PhANS* were blasted with *N. tabacum* EST database. SYBR^®^ Premix Ex Taq from TaKaRa was used for qPCR with an Applied Biosystems 7500 real-time PCR system (Applied Biosystems). The tobacco gene *EF1α* was used as an internal control (Zhang et al., 2009) in all qPCR reactions. Three biological replicates were performed for each experiment.

### Photometric Determination of Anthocyanins

Petals of mature flowers were harvested, ground in liquid nitrogen to produce a fine powder, and then immediately freeze-dried, and stored at −80°C until use. Anthocyanins were detected as previously described (Zhang et al., 2009). All samples were measured as triplicates in three independent biological replicates. Error bars represent +SE.

### Yeast Two-Hybrid Screen

We cloned the cDNA sequences of *CsTRY* (full-length) and fused it into the pGADT7 vectors. The ORF of *CsAN1*, a homolog gene of the *Arabidopsis PhANTHOCYANIN1* (*AN1*) (Spelt, 2000) was cloned and fused into the pGBKT7. All recombinant constructs were separately transformed into the yeast strain AH109. At least three independent experiments were performed, and the result of one representative experiment is shown.

### Scanning Electron Microscopy

*N. tabacum* young leaf samples were fixed, washed, post fixed, dehydrated, coated (Chen et al., 2014), and observed using a Hitachi S-4700 scanning electron microscope with a 2-kV accelerating voltage.

### Bimolecular Fluorescence Complementation (BiFC) Assay

To generate the BiFC constructs, the full-length cDNA sequences of *CsTRY* and *CsAN1* were cloned and fused with the pXY104 and pXY106 vectors (Yu et al., 2008; Liu and Howell, 2010). Tobacco (*N. tabacum*) leaves were used for co-expression studies as previously described (Schütze et al., 2009). The fluorescence signal was detected 2 to 4 days after infiltration, using an Olympus BX 51 fluorescence microscope to acquire fluorescent images. YFP (yellow fluorescent protein) imaging was performed at an excitation wavelength of 488 nm. CFP served as the internal control in all BiFC analyses. At least three independent replicates were performed, and the result of one representative experiment is shown.

### Metabolite Profiling

The petals of transgene plants were grounded into a fine powder. Each 20 mg of fine powder was used for metabolite extraction prior to UHPLC-Q-TOF-MS analysis. The metabolite extraction was analyzed as previously described (Hu et al., 2018). The metabolites were annotated by searching the Personal Compound Database and Library (PCD/PCDL) (Hu et al., 2015), and by comparing the MS and MS/MS of the compounds in the Metlin database (Want, 2005) and the Massbank database (Horai et al., 2010). Data acquisition, metabolite annotation and peak area extraction were performed with the Agilent softwares (Agilent Technologies Inc., Palo Alto, CA, USA), of MassHunter Acquisition 7.0, MassHunter Qualitative 7.0 and Mass Profinder 8.0, respectively. All measurements were performed in three replicates per genotype.

## Results

### 1. Flower Pigmentation and Trichome Distribution Were Affected in Transgenic Tobacco Plants Overexpressing *CsTRY*, but Not in Plants Overexpressing *CsMYB6*

**Figure 1:**
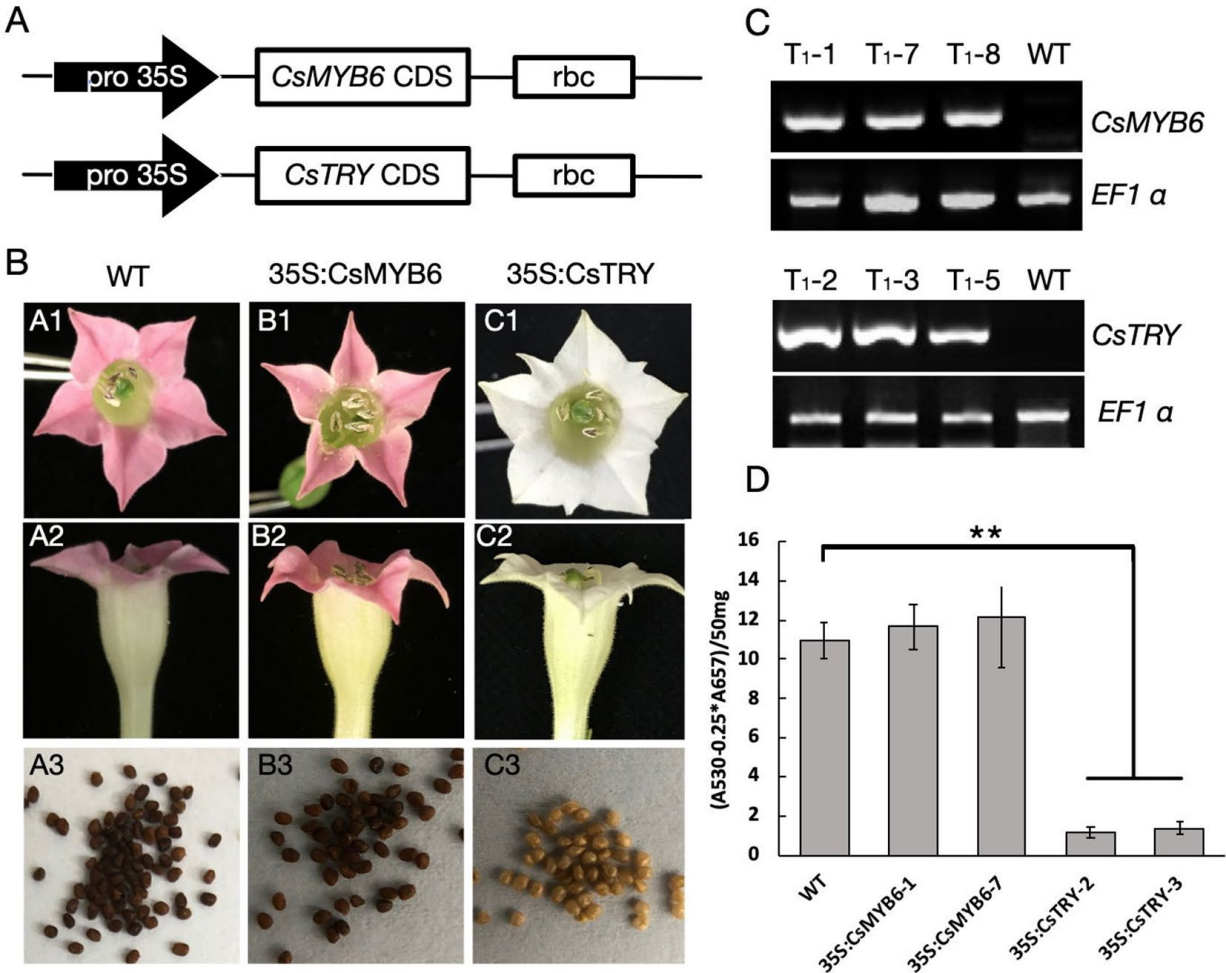
The gene construct for ectopic expression of *CsTRY* and *CsMYB6* and changes in flower and seed pigmentation phenotype of transgenic tobacco. **(A)** Structure of the gene construct used to express. **(B)** *CsTRY* and *CsMYB6* expressing lines exhibiting different phenotype characteristics. A1–A3 is the petal and seeds of WT; B1–B3 is the petal and seeds of 35S:CsMYB6 transgenic tobacco plants; and C1–C3 is of 35S:CsTRY transgenic plants **(C)** Semi-quantitative RT-PCR analysis of *CsTRY* and *CsMYB6* expression levels in mature leaves of T1 generation plants. EF1α transcript abundance was used as a control. **(D)** Photometric determination of anthocyanin content in methanolic extracts of petals in tobacco lines *35S:CsTRY* (35S:CsTRY-2, 35S:CsTRY-3), *35S:CsMYB6* (35S:CsMYB6-1, 35S:CsMYB6-7) and the wild-type. A_530_, absorption at 530 nm; A_657_, absorption at 657 nm. Error bars represent +SE. Significant differences were determined according to Duncan’s multiple range test (P < 0.05) or Student’s t-test (**P < 0.01).

To explore the function of *CsTRY* and *CsMYB6*, we used 35S promoter to regulate these two genes and overexpress them in tobacco by genetic transformation (**Figure 1A**). In transgenic 35S:CsTRY tobacco, there were clear phenotypic changes in petal pigmentation, which resulted in a complete loss of pigmentation and pure white petals. Moreover, there was a decrease in seed pigmentation, which was lighter than the wild type (**Figure 1B**).

Anthocyanin quantification results measured by spectrophotometer revealed that anthocyanin accumulation in the petals of 35S:CsTRY transgenic tobacco plants was clearly reduced, indicating that *CsTRY* may be negatively regulating the synthesis of tobacco anthocyanin (**Figure 1D**).

**Figure 2:**
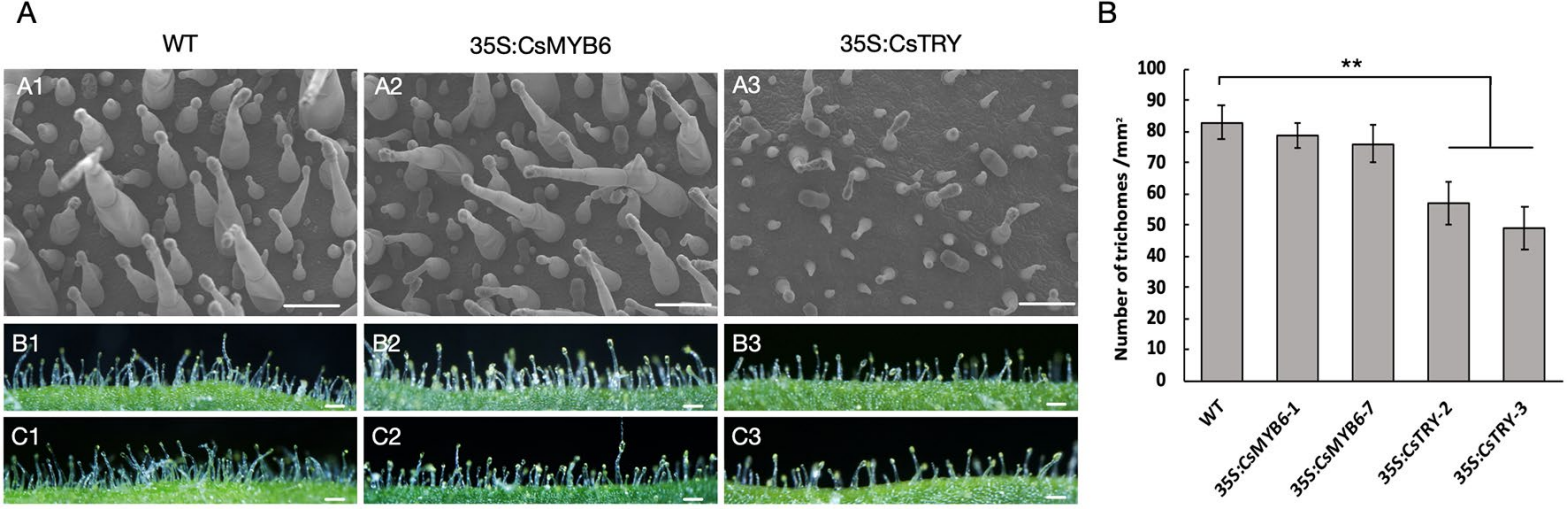
The morphology and quantity of trichome in wild type and 35S:CsTRY, 35S:CsMYB6 tobacco lines **(A)** Patterns of trichome distribution on the leave surface of T1 generation plants of *CsTRY* and *CsMYB6* overexpression transgenic tobacco. Bars = 100 µm. **(B)** Number of trichomes on 1 mm^−2^ adaxial surface of the leave of T1 generation plants of *CsTRY* and *CsMYB6* overexpression transformed lines and the wild-type. Error bars represent +SE. Significant differences were determined according to Duncan’s multiple range test (P < 0.05) or Student’s t-test (**P < 0.01).

In addition, the morphology and quantity of glandular hairs of transgenic lines were observed by scanning election microscopy (**Figure 2**). It was found that the number of long stalked glandular hairs and the density of glandular hairs decreased, which is consistent with the known negative regulation of the epidermis. However, overexpression of *CsMYB6* in tobacco showed no difference in phenotypes, such as flower pigment and glandular density.

### 2. *CsTRY* Negatively Regulates the Synthesis of Anthocyanins by Suppressing the Expression of Genes in the Flavonoid Metabolic Pathway

**Figure 3:**
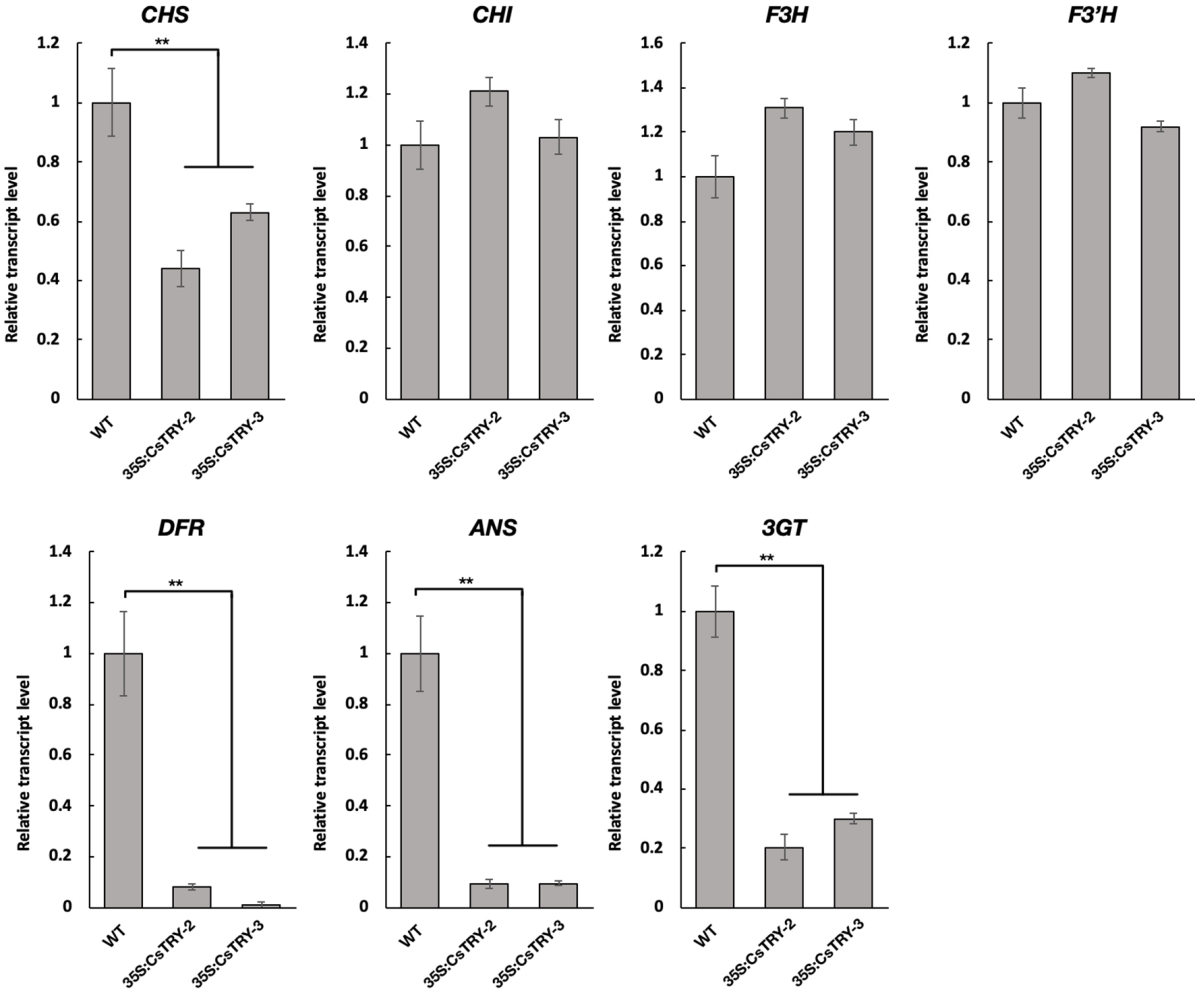
Relative transcript levels of flavonoid structural genes in the petals of tobacco lines *35S:CsTRY* (35S:CsTRY-2, 35S:CsTRY-3) and wild type, with the *EF1α* gene as an internal control. Error bars represent +SE. Significant differences were determined according to Duncan’s multiple range test (P < 0.05) or Student’s t-test (**P < 0.01).

To elucidate the molecular mechanisms involved in the marked decrease of anthocyanins in 35S:CsTRY transformants, the transcript levels of seven key genes encoding the enzymes of the flavonoid pathway from the first stage to the third were measured in flowers by real-time RT-PCR analyses (**Figure 3**).

Based on the different responses of the seven genes to *CsTRY*, we divided them into three categories. The first type is Chalcone synthase (CHS) located upstream of the second stage of the flavonoid metabolic pathway, and its expression level is significantly inhibited; the second type contains Chalcone isomerase (CHI), flavanone 3-hydroxylas (F3H), and flavonoid 3′-hydroxylase (F3′H) located downstream of the second stage of the flavonoid metabolic pathway, and its expression level is upregulated to varying degrees. The third category is dihydroflavonol4-reductas (DFR), Anthocyanidin Synthase (ANS) and Anthocyanin 3-0-g1ucosyltransferase (3GT), which is directly related to the synthesis of anthocyanins and is clearly strongly inhibited.

**Figure 4:**
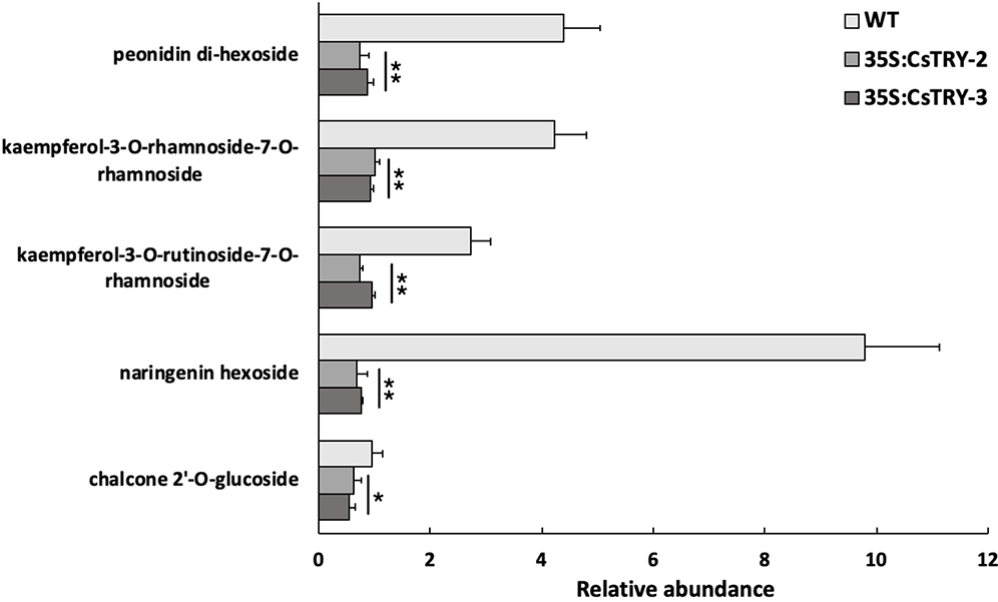
Determination of the relative abundance of flavonoids in flowers of *CsTRY* overexpressing transgenic tobacco lines (35S:CsTRY-2, 35S:CsTRY-3). Error bars represent +SE. Significant differences were determined according to Duncan’s multiple range test (P < 0.05) or Student’s t-test (*P < 0.05, **P < 0.01).

Furthermore, we detected a variety of flavonoids in transgenic tobacco petals by LC-MS (**Figure 4**). We found that a number of secondary metabolites related to anthocyanins, such as (kaempferol-3-O-rhamnoside-7-O-rhamnoside and kaempferol-3-O-rutinoside-7-O-rhamnoside), naringenin hexoside, chalcone 2″-O-glucoside, anthocyanin (peonidin di-hexoside I, II, and III), were significantly reduced in transgenic plants.

This is consistent with the expression of genes. In the secondary stage of Anthocyanin synthesis, *CHS* catalyzes the stepwise condensation of three acetate units from malonyl-COA with p-coumaroyl-COA to yield tetrahydroxychalcone. *CHI* then catalyzes the stereospecific isornerization of the yellow-colored tetrahydroxychalcone to the colorless naringenin. Naringenin is converted to dihydrokaempferol by *F3H*. Thus, the decrease in the expression of *CHS* and the increase in the expression of *CHI, F3H* reduced the content of chalcone and trihydroxyflavanone. In the last stage of Anthocyanin synthesis, the dihydroflavonol is reduced to the flavan 9,4 cis-diol (leucoanthocyanidins) by dihydroflavone-4-reductase (DFR). Then Anthocyanin formed by catalysis of *ANS* and *3GT*. Owing to the inhibited of the expression three genes, *DFR, ANS,* and *3GT*, the decrease of Anthocyanin is reasonable.

### 3. The CsTRY Protein Interacts With the Known Anthocyanin Synthesis Regulator *CsAN1*

**Figure 5:**
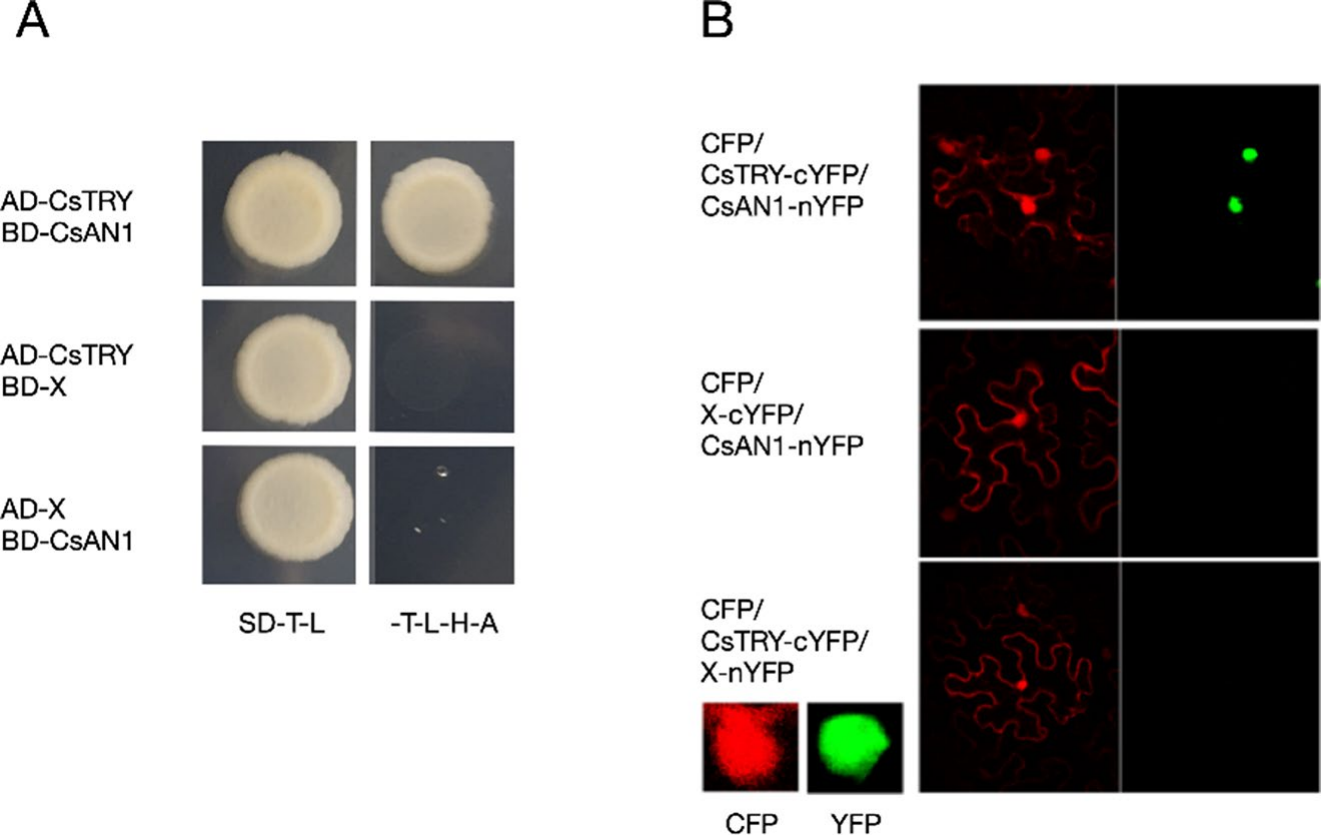
Yeast two-hybrid analysis and BiFC **(A)** Activation activity of CsTRY to CsAN1 in yeast. **(B)** BiFC analysis of the physical interaction between CsAN1 (fused with the N-terminal fragment of YFP) and CsTRY (fused with the C-terminal fragment of YFP).

The PhAN1 protein has previously been shown to be essential for anthocyanin synthesis in petunia. To further verify whether CsTRY can interact with known modulators of anthocyanin metabolism, we cloned the homologous gene of *PhAN1* in cucumber, *CsAN1*, through yeast two-hybrid and BiFC and found that CsTRY can interact with CsAN1 (**Figure 5**).

## Discussion

Trichomes are generally considered biofactories that produce secondary metabolites. The genes regulating unicellular trichome developments are usually related to anthocyanin synthesis (Jin and Martin, 1999). In *A. annua*, which has multicellular trichomes, genes involved in the development of trichomes also regulate the synthesis and transportation of secondary metabolites, such as artemisinin (Yan et al., 2018). In the cucumber trichome development mutants *csgl3/tril* and *csgl1/mict/tbh*, the genes that regulate anthocyanin synthesis are also differentially expressed, suggesting that although trichomes morphology and related genes differ between multicellular and unicellular trichomes, the mechanism of secondary metabolites coupled with epidermal hair development is conserved. Overexpression of *CsTRY* in tobacco can affect the flowering and seed coat color, but overexpression of *CsMYB6* does not. This result suggests that although both genes can regulate the density of trichomes in cucumber, the specific mechanism and range of the regulation may not be the same. In addition, *CsMYB6* was significantly downregulated in the cucumber hairless mutants *tril* and *mict*, but the expression of *CsTRY* did not change, which also indicated that the regulation patterns of *CsTRY* and *CsMYB6* may be different (Chen et al., 2014; Li et al., 2015; Zhao et al., 2015).

In previous study, overexpression *CsTRY* or *CsMYB6* in cucumber can decrease the density of fruit trichome and *CsTRY* is directly regulated by *CsMYB6*. However, overexpression of CsMYB6 inhibited rather than promoted, the expression of *CsTRY*, which means the relationship between *CsTRY* and *CsMYB6* is not simple (Yang et al., 2018). In this study, overexpression of *CsTRY* in tobacco can affect the number of trichome, but overexpression of *CsMYB6* does not. It can be inferred that *CsTRY*, a R3 MYB transcription factor in the relative downstream, was more directly related to glandular trichomes and metabolites, while *CsMYB6*, a R2R3 MYB transcription factor in the relative upstream, might be affected by other proteins in tobacco. Interestingly, overexpression *CsTRY* or *CsMYB6* in cucumber can decrease the density of fruit trichome rather than other organs (Yang et al., 2018), which means the regulation mechanism of fruit trichome is different from that of other organs. *SlMIXTA-like*, a R2R2MYB transcription factor of tomato, regulates trichome formation on fruit surface, which also indicates that the formation of fruit trichome may be different from that of other organs (Ying et al., 2019). Therefore, overexpress *CsMYB6* in tobacco did not change the phenotype, suggesting that *CsMYB6* only plays a role in the regulation of cucumber fruit trichome, not other organs.

At present, there are three main ways to regulate anthocyanin synthesis: MYB-bHLH protein binary complex, MYB-WD40 protein binary complex, which is independent of bHLH transcription factor, and MYB-bHLH-WD40 protein ternary complex. The anthocyanin pathway in most plants is activated by MYB-bHLH-WD40 ternary complex (Broun, 2005; Antoine et al., 2010). In this study, we found that CsTRY can interact with the bHLH protein CsAN1. *AN1* is an important regulator involved in anthocyanin synthesis in *Petunia hybrida*. It is a homology of the structural gene DFR and can directly regulate the expression of *DFR* (Spelt, 2000). Without the research of WD40 protein, we speculate that *CsTRY* may function by forming a ternary complex of MYB-bHLH-WD40 protein or a binary complex of MYB-bHLH protein in tobacco. Moreover, *CsTRY* can interact with *CsAN1*, a homolog of the *Arabidopsis AN1* gene, indicating that it also acts as a negative regulator of anthocyanin synthesis in cucumber and has regulatory mechanisms similar to *Arabidopsis*.

In this study, the overexpression of *CsTRY* in tobacco greatly affected the synthesis of anthocyanin, indicating that *CsTRY* may have conserved function in cucumber. A number of transcription factors (*e.g.,* *CsGl1/Mict*/*tbh*, *CsGl3/Tril*, *Tu* and *Ts*) play key roles in cucumber trichome (spine) differentiation and development. However, the functions of their homologues in *Arabidopsis* are irrelevant to trichome development (Yang et al., 2014; Zhao et al., 2015; Guo et al., 2018). Moreover, *CsTRY* can complement the *Arabidopsis try* mutant, whereas *CsMYB6* cannot complement the *gl1* mutant. These results suggest that the regulation network of multicellular-trichome development may be partially consistent with unicellular-trichome development, but key genes, such as *CsGl3/Tril* and *CsGl1/Mict/tbh*, may have independent evolutionary pathways in the two different trichome types.

## Data Availability Statement

All datasets generated for this study are included in the manuscript/supplementary files.

## Author Contributions

HH conceptualized the research. JuP designed experiments. JiP participated in writing, editing, and revising the manuscript. RC conceptualized the research, designed and performed experiments. GW performed experiments and prepared figures. LZ performed experiments, analyzed the data, and wrote the manuscript. HD prepared the figures.

## Funding

This work was supported by the National Key R&D Program of China (Grant No. 2018YFD0100701), National Natural Science Foundation of China (31471156), Shanghai Agriculture Applied Technology Development Program (Grant No.G2015060402).

## Conflict of Interest

The authors declare that the research was conducted in the absence of any commercial or financial relationships that could be construed as a potential conflict of interest.
